# Adipose-Derived Mesenchymal Stem Cell Osteodifferentiation after Exposure to Beta-Tricalcium Phosphate Bioceramic Granules with 300 to 600 and 600 to 1,000 µm Sizes

**DOI:** 10.1055/s-0045-1806964

**Published:** 2025-05-07

**Authors:** Pamela Handy Cecilia, Ida Bagus Narmada, Rini Devijanti Ridwan, Diah Savitri Ernawati, Taufan Bramantoro, Devi Rianti, Khairul Anuar Shariff, Wibi Riawan, Putri Cahaya Situmorang, Alexander Patera Nugraha

**Affiliations:** 1 Doctoral Program of Dental Medicine, Faculty of Dental Medicine, Universitas Airlangga, Surabaya, Indonesia; 2 Department of Orthodontics, Faculty of Dental Medicine, Universitas Airlangga, Surabaya, Indonesia; 3 Department of Oral Biology, Faculty of Dental Medicine, Universitas Airlangga, Surabaya, Indonesia; 4 Department of Oral Medicine, Faculty of Dental Medicine, Universitas Airlangga, Surabaya, Indonesia; 5 Department of Dental Public Health, Faculty of Dental Medicine, Universitas Airlangga, Surabaya, Indonesia; 6 Department of Dental Material, Faculty of Dental Medicine, Universitas Airlangga, Surabaya, Indonesia; 7 School of Materials and Mineral Resources Engineering, Universiti Sains Malaysia, Nibong Tebal, Penang, Malaysia; 8 Department of Biomolecular Biochemistry, Faculty of Medicine, Universitas Brawijaya, Malang, Indonesia; 9 Biology Study Program, Department of Biology, Faculty of Mathematics and Natural Sciences, Universitas Sumatera Utara, Medan, Indonesia

**Keywords:** β-TCP, adipose-derived mesenchymal stem cells, medicine, regenerative medicine, tissue engineering

## Abstract

**Objective:**

Beta-tricalcium phosphate (β-TCP) is a synthetic graft material with excellent biocompatibility, osteoconductivity, and osteoinductivity. β-TCP may induce adipose-derived mesenchymal stem cells (ADMSCs) osteodifferentiation. This study aims to investigate the osteoinductivity of 300 to 600 and 600 to 1,000μm β-TCP in ADMSCs.

**Materials and Methods:**

ADMSCs were obtained from the visceral adipose tissue of young male rabbits. To determine the osteoinductive ability, bone morphogenic protein 2 (BMP-2), Osterix, runt-related transcription factor 2 (Runx2), alkaline phosphatase (ALP), osteopontin, and osteonectin expression was examined using an immunochemical assay on ADMSCs conditioned with an osteogenic medium and a β-TCP bioceramic with granule sizes of 300 to 600 and 600 to 1,000 µm (100 ng diluted to 100 nmol as the final concentration). A 3,3′-diaminobenzidine staining kit was used for immunocytochemical staining. Anti-BMP-2, anti-Osterix, anti-Runx2, anti-ALP, anti-osteopontin, and anti-osteonectin monoclonal antibodies were employed at a 1:500 dilution. A light microscope with magnifications of 400× and 1,000× was used to manually observe and examine cultures in five different fields of view.

**Results:**

BMP 2, Runx2, Osterix, and ALP expression was higher in ADMSCs + β-TCP 300 to 600 µm compared with the control group ( *p* < 0.05). Osteonectin and osteopontin expression was higher in ADMSCs + 300 to 600 µm β-TCP compared with the control group ( *p* < 0.05) and ADMSCs + 600 to 1,000 µm β-TCP ( *p* < 0.05).

**Conclusion:**

ADMSC osteodifferentiation was influenced by β-TCP bioceramic granule size. The considerable difference in osteonectin and osteopontin expression supports the idea that 300 to 600 µm β-TCP induce ADMSCs osteodifferentiation than 600 to 1,000 µm β-TCP.

## Introduction

Cleft lip and/or palate (CL/P) typically have oral health issues as a result of anomalies in the alveolar process. 1 Alveolar process bone loss is substantial in people with CL/P. For alveolar cleft regeneration in CL/P, regeneration of the alveolar defect is therefore necessary. 2 The effectiveness of alveolar bone grafting (ABG), a crucial technique in the overall therapy of patients with CL/P, significantly influences orthognathic surgical treatment. 3

For autologous ABG, ilium is the most often utilized tissue donor. The ilium can be used as a donor site, although patients may experience pain, ureteral injuries, avulsion fractures, hematomas, infections, herniation of abdominal contents, abnormalities in gait, cosmetic deformities, sacroiliac joint violations, and neurovascular injury. Thus, the use of allografts, xenografts, and synthetic grafts has been investigated as alternative options to replace the autologous iliac crest in ABG. 1 4

One synthetic graft material that has been tested in clinical studies as a bone substitute is beta-tricalcium phosphate (β-TCP), which exhibits a high rate of new bone creation after being transplanted into a bone injury. 5 Excellent biocompatibility, osteoconductivity, and osteoinductivity are all displayed by β-TCP, which has also been demonstrated to promote mesenchymal cell differentiation and proliferation. 5 6 Furthermore, β-TCP exhibits a compressive strength comparable to cancellous bone when resorption takes place through phagocytic, enzymatic, and hydrolysis processes. 7

The β-TCP scaffold structure can undergo very quick osteotransduction in the trabecular structure through a combination of osteoclast-mediated resorption and osteoconduction. 5 Comparing β-TCP to other synthetic graft materials, such as hydroxyapatite, its benefit is that the body can break it down at a rate that is comparable to the rate of new bone growth. This allows for the creation of bone with uniform elasticity and lowers the risk of fracture. 8

It has been observed in research that β-TCP can more effectively produce new bone when combined with growth factors or cells. 7 9 10 Nevertheless, limited studies have documented how adipose-derived mesenchymal stem cells (ADMSCs)—a classification of mesenchymal stem cells (MSCs) that aid in alveolar bone regeneration—respond behaviorally to the delivery of β-TCP bioceramic granules of different sizes. ADMSCs are MSCs that have been separated from adipose tissue. Because they are easily accessible and simple to extract, they are one of the most popular sources of MSCs, along with bone marrow. Regenerative medicine greatly benefits from ADMSCs' fibroblast-like morphology, spindle-shaped form, strong proliferation activity, high potential for multilineage differentiation, and immunomodulatory activity. 11 12 ADMSCs can differentiate osteogenically, which is similar to MSCs derived from bone marrow, but they proliferate at a greater rate than MSCs derived from bone marrow. 12 13 Furthermore, because of their widespread availability and simplicity of isolation, ADMSCs have drawn much attention due to their potential for bone regeneration. 14

Endogenous MSCs must undergo osteogenic differentiation to promote bone regeneration. A member of the transforming growth factor β class, bone morphogenetic protein 2 (BMP-2) is a growth factor. It has been demonstrated via considerable research that BMP-2 plays a critical role in the bone remodeling process. BMP-2 stimulates MSCs to develop into osteoblasts when the bone needs to be remodeled. 15 16 Runt-related transcription factor 2 (Runx2) and Osterix (Osx) are osteogenic genes that are essential for the development of preosteoblasts into dormant osteoblasts, and BMP-2 can activate these genes. 16 17 As evidenced by the presence of several markers, including osteonectin and alkaline phosphatase (ALP), inactive osteoblasts will eventually turn into active ones. Afterward, the active osteoblasts will become osteocytes and be entrenched in the bone. The existence of indicators such as osteopontin demonstrates this. 17 18 Studies reveal that the size of the β-TCP material has a major effect on its osteoinductive properties and biological response. 5 To identify the ideal size of β-TCP as a bone graft material, this study examined two sizes of β-TCP bioceramic granules (300–600 and 600–1,000 µm). The selection of the two β-TCP sizes was based on the market-available particle sizes and was tested over time. Furthermore, prior studies have demonstrated that the activity of bone cells can be considerably impacted by macro- and micropores in β-TCP that range in size from 300 to 600 µm. 19 20

This study hypothesized that a 300- to 600-µm β-TCP bioceramic may enhance the osteodifferentiation of ADMSCs through BMP-2, Runx2, Osx, ALP, osteonectin, and osteopontin. Based on the above information, this study used immunocytochemistry to examine several key biomarkers during the osteodifferentiation process, including BMP-2, Runx2, Osx, ALP, osteonectin, and osteopontin, to assess the osteoinductivity of β-TCP bioceramic granules with sizes of 300 to 600 and 600 to 1,000 µm on the osteogenic differentiation of ADMSCs *in vitro* .

## Material and Methods

### Study Design and Ethical Clearance

A simple double-blind randomized true experimental design with a posttest-only control group was the study design used in this investigation. The Faculty of Dental Medicine at Universitas Airlangga granted ethical clearance for this study under the number 682/HRECC.FODM/IX/2022.

### ADMSC Isolation and Culture

The visceral fat tissue of young male rabbits was used to identify ADMSCs. Using a light microscope, the morphology of the ADMSCs was examined. An inverted fluorescent microscope (Leica, United States) was used to view the immunocytochemical analysis with green immunofluorescence staining using cluster of differentiation (CD) 73, CD90, CD105, and CD45 (polyclonal antibodies from Santa Cruz, United States) at a magnification of 100 × . 2 13 21

### Osteoinductivity of β-TCP Granules in ADMSC Osteogenic Differentiation

To determine the osteoinductive ability, the expression of BMP-2, Osx, Runx2, ALP, osteopontin, and osteonectin was examined using an immunochemical assay on an ADMSC-conditioned osteogenic medium (Osteomax, Merck, United States) and β-TCP bioceramics obtained from School of Materials and Mineral Resources Engineering, Universiti Sains Malaysia, Nibong Tebal, Penang, Malaysia with granule sizes of 300 to 600 and 600 to 1,000 µm (100 ng diluted to 100 nmol as the final concentration). A 3,3′-diaminobenzidine staining kit (Sigma-Aldrich) was used for immunocytochemical staining. BMP-2, Osx, Runx2, ALP, osteopontin, and osteonectin monoclonal antibodies (AbMo from Abcam, United States) were employed at a 1:500 dilution. A light microscope (Olympus, Japan), with magnifications of 400× and 1,000 × , was used to manually observe and examine the cultures in five different fields of view. 16 17 18

### Data Statistical Analysis

The study data were recapitulated and analyzed using means and standard deviations (SDs) in Statistical Software for Social Science (SPSS) version 27.0 (IBM Corporation, Chicago, Illinois, United States). One-way analysis of variance (ANOVA) and post hoc Tukey's honest significant difference (HSD) were performed for normal and homogeneous data, and Kruskal–Wallis and Mann–Whitney tests were performed for abnormal and nonhomogeneous data. The level of statistical significance was set at *p* < 0.05. Asterisks (*) indicated the degree of statistical significance (* *p* < 0.05), while “ns” represented nonsignificant results ( *p* > 0.05).

## Results

**Fig. 1:**
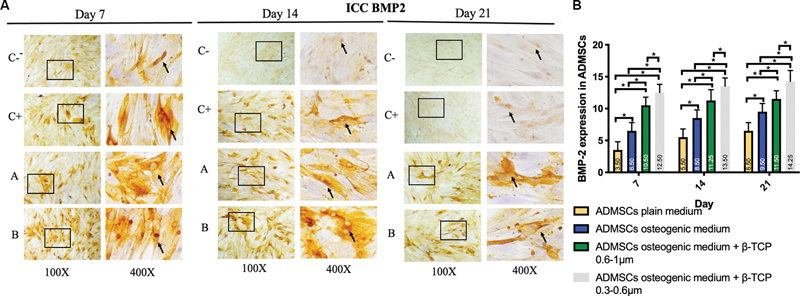
( **A** ) Positive bone morphogenic protein 2 (BMP-2) expression in brown color (black square and arrow) in (C − ) adipose-derived mesenchymal stem cell (ADMSC) on plain medium; (C + ) ADMSC on osteogenic medium; (A) ADMSC on osteogenic medium + 600–1,000-µm beta-tricalcium phosphate (β-TCP); (B) ADMSC on osteogenic medium + 300–600-µm β-TCP observed via light microscopy at 100× and 400× magnification. ( **B** ) Average and standard deviation graphical bars of BMP-2 expression in each group. *Significant difference between groups at *p* < 0.05.

**Fig. 2:**
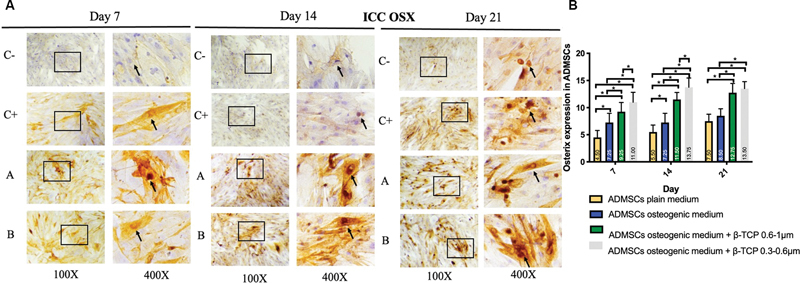
( **A** ) Positive Osterix (OSX) expression in brown color (black square and arrow) in (C–) adipose-derived mesenchymal stem cell (ADMSC) on plain medium; (C + ) ADMSC on osteogenic medium; (A) ADMSC on osteogenic medium + beta-tricalcium phosphate (β-TCP) 600–1,000 µm; (B) ADMSC on osteogenic medium + β-TCP 300–600 µm observed via light microscopy at 100× and 400× magnification. ( **B** ) Average and standard deviation graphical bars of Osterix expression in each group. *Significant difference between groups at *p* < 0.05.

**Fig. 3:**
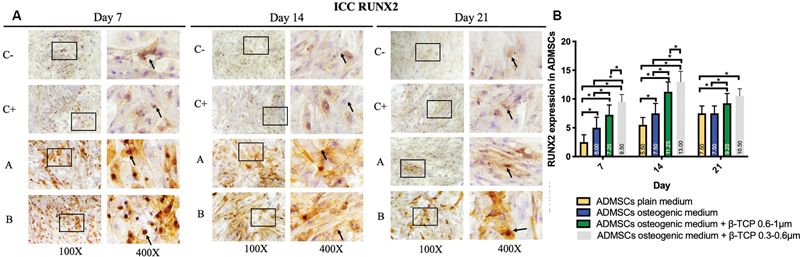
( **A** ) Positive runt-related transcription factor 2 (Runx2) expression in brown color (black square and arrow) in (C–) adipose-derived mesenchymal stem cell (ADMSC) on plain medium; (C + ) ADMSC on osteogenic medium; (A) ADMSC on osteogenic medium + beta-tricalcium phosphate (β-TCP) 600–1,000 µm; (B) ADMSC on osteogenic medium + β-TCP 300–600 µm observed via light microscopy at 100× and 400× magnification. ( **B** ) Average and standard deviation graphical bars of Runx2 expression in each group. *Significant difference between groups at *p* < 0.05.

**Fig. 4:**
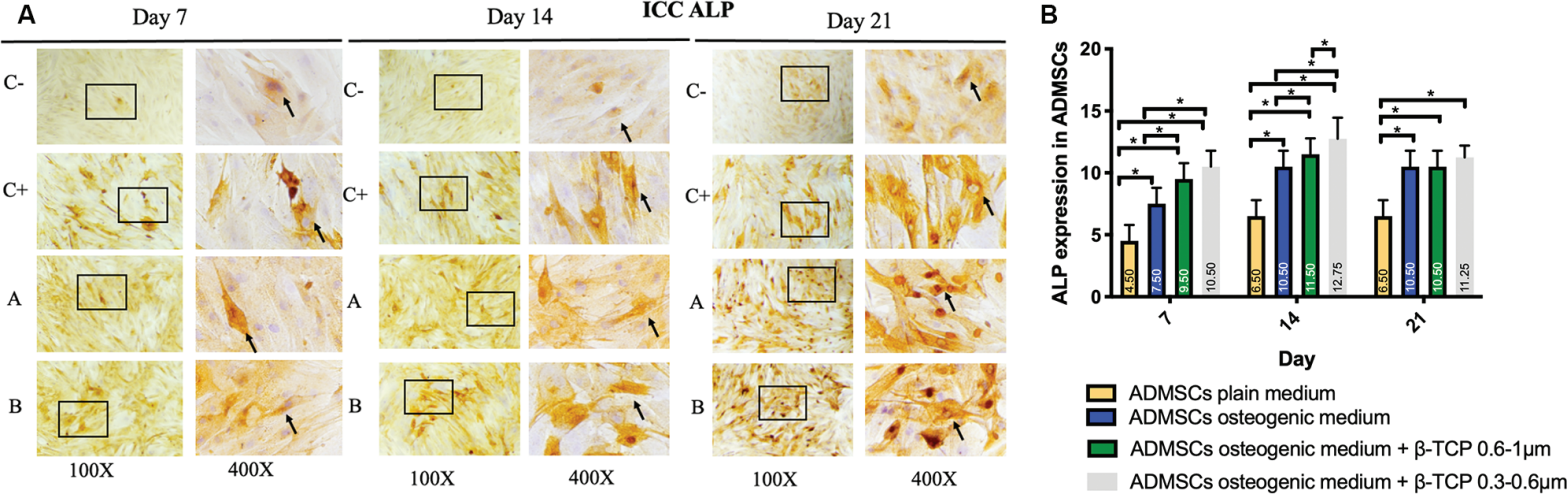
( **A** ) Positive alkaline phosphatase (ALP) expression in brown color (black square and arrow) in (C–) adipose-derived mesenchymal stem cell (ADMSC) on plain medium; (C + ) ADMSC on osteogenic medium; (A) ADMSC on osteogenic medium + beta-tricalcium phosphate (β-TCP) 600–1,000 µm; (B) ADMSC on osteogenic medium + β-TCP 300–600 µm observed via light microscopy at 100× and 400× magnification. ( **B** ) Average and standard deviation graphical bars of ALP expression in each group. *Significant difference between groups at *p* < 0.05.

**Fig. 5:**
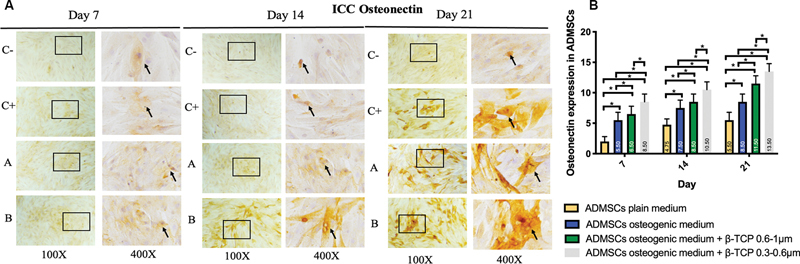
( **A** ) Positive osteonectin expression in brown color (black square and arrow) in (C–) adipose-derived mesenchymal stem cell (ADMSC) on plain medium; (C + ) ADMSC on osteogenic medium; (A) ADMSC on osteogenic medium + beta-tricalcium phosphate (β-TCP) 600–1,000 µm; (B) ADMSC on osteogenic medium + β-TCP 300–600 µm observed via light microscopy at 100× and 400× magnification. ( **B** ) Average and standard deviation graphical bars of osteonectin expression in each group. *Significant difference between groups at *p* < 0.05.

**Fig. 6:**
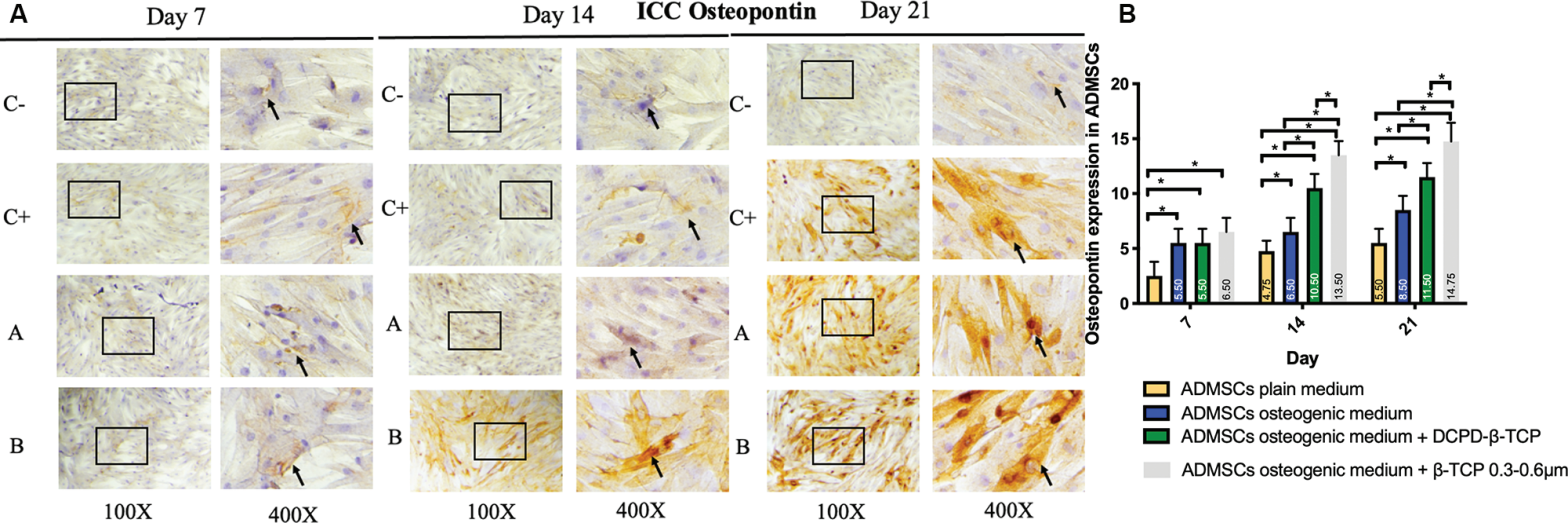
( **A** ) Positive osteopontin expression in brown color (black square and arrow) in (C–) adipose-derived mesenchymal stem cell (ADMSC) on plain medium; (C + ) ADMSC on osteogenic medium; (A) ADMSC on osteogenic medium + beta-tricalcium phosphate (β-TCP) 600–1,000 µm; (B) ADMSC on osteogenic medium + β-TCP 300–600 µm observed via light microscopy at 100× and 400× magnification. ( **B** ) Average and standard deviation graphical bars of osteopontin expression in each group. *Significant difference between groups at *p* < 0.05.

The study data were found to be normally distributed based on the one-sample Kolmogorov–Smirnov test, and the variances of all the data were homogeneous, according to the Levene test ( *p* > 0.05). For the BMP-2, Osx, Runx2, ALP, osteonectin, and osteopontin expression on days 7, 14, and 21, an ANOVA test followed by Tukey's HSD was conducted ( *p* < 0.05). The expression of BMP-2, Osx, Runx2, ALP, osteonectin, and osteopontin was observed in the ADMSC-positive and -negative groups, ADMSCs + 300 to 600 µm β-TCP group, and ADMSCs + 600 to 1,000 µm β-TCP group, with a brown color indicating positive expression in each biomarker, observed at magnifications of 100× and 400× using a light microscope ( Figs. 1A – 6A ). Figs. 1B – 6B present the means and SDs of BMP-2, Osx, Runx2, ALP, osteonectin, and osteopontin expression in each group.

The results showed that the administration of β-TCP with a size of 600 to 1,000 µm or β-TCP with a size of 300 to 600 µm could increase osteogenic differentiation in the ADMSCs, which was indicated by a significant increase in the expression of BMP-2, Runx2, Osx, ALP, osteonectin, and osteopontin compared with the ADMSCs that were not given β-TCP ( *p* = 0.001; *p* < 0.05). In addition, the ADMSCs given β-TCP with a size of 300 to 600 µm showed better osteogenic differentiation compared with the ADMSCs given β-TCP with a size of 600 to 1,000 µm, although statistically, this was only significant for the osteopontin and osteonectin markers of β-TCP ( *p* = 0.001; *p* < 0.05) and was not significant for the BMP-2, Osx, Runx2, and ALP markers of β-TCP ( *p* < 0.05).

## Discussion

β-TCP is a substance that is frequently utilized in dentistry and medicine because of its good osteoconductive, biocompatible, bioresorbable, and bioactive qualities. The bioresorbability of β-TCP makes it a popular bone substitute. 2 9 10 Previous studies have demonstrated that β-TCP is biologically destroyed well by the body due to its resorption rate, which is directly proportional to the rate at which new bone is formed. In β-TCP, micropores with sizes between 0.1 and 100 µm aid in osteoinduction and enhanced resorption. 22 Mohammed et al's investigation revealed that β-TCP develops macro- and micropores that range in size from 300 to 600 µm. 22 Since they can enhance the regulation of adhesion, proliferation, and cell differentiation, macro- and micropores in the scaffold have a major effect on the activities of bone-like cells, increasing the scaffold's rate of degradation within the body. 22 23

The ADMSCs employed in this investigation were extracted from male rabbits' visceral tissue. Following isolation, green immunofluorescence labeling was used for immunocytochemical analysis to confirm that the isolated cells were ADMSCs. An ADMSC is defined by the International Society for Cellular Therapy (2006) as follows: (1) the cell must be attached to plastic, (2) it must express CD73, CD90, and CD105 but not CD45, CD34, CD14 or CD11b, CD79a or CD19, or human leukocyte antigen DR isotype surface molecules, and (3) it must be able to be differentiated into preadipocytes, chondrocytes, and osteoblasts. 11 13

This work used six bone biomarkers—BMP-2, Runx2, Osx, ALP, osteonectin, and osteopontin—to check the effect of β-TCP on the osteogenic differentiation of ADMSCs. The osteoblastogenesis process served as the basis for the selection of the six biomarkers, each of which contributes to the complete osteoblast creation process, from the earliest to the last phases. Endogenous mesenchymal cells that have intermediate BMP-2 will develop into preosteoblasts during the early phases of osteoblastogenesis. At this point, there will be an increase in the expression of Osx and Runx2. 15 Additionally, preosteoblasts will develop into dormant osteoblasts, which are identified by an elevated expression of ALP. 17 Inactive osteoblasts will then undergo differentiation to become active osteoblasts. An increase in osteonectin expression will be observed at this point. Additionally, the tissue will receive osteocytes, crucial in promoting mineralization, which is indicated by an increase in the osteopontin biomarker. 17 18

One BMP component that acts as an osteoinductive agent is BMP-2. It is yet unclear how β-TCP injection causes BMP-2 to be upregulated. 24 Chen et al discovered that β-TCP can increase macrophage BMP-2 expression, indicating a connection to inflammation. Osteoblasts grow from mesenchymal progenitor cells when BMP-2 activates the receptor and sends signals via the suppressor of mothers against decapentaplegic (SMAD)-dependent and SMAD-independent pathways. BMP-2, which is produced by macrophages, aids in osteogenesis during bone regeneration. By attaching itself to bone morphogenic protein receptor-2, the released BMP-2 can activate SMAD5. Afterward, the activated SMAD5 and SMAD4 can combine to generate a molecule that is carried into the nucleus and stimulates the synthesis of distal-less homeobox 5 (Dlx5). It has been demonstrated that Dlx5 induces Runx2, which causes MSCs to differentiate osteogenically. 25

In the development of MSCs, BMP-2 has a special function that guides the transition of progenitor cells into Runx2 and Osx activation cells. It has been shown that BMP-2 increases Osx expression by activating Runx2 through SMAD and Runx2 signaling. Runx2 modifies Osx promoter activity by directly binding to the Osx promoter region. One new gene candidate for osteogenic development is nuclear factor I-C (NfIc), which is expressed in human osteoblasts and osteoblast-like cell lines. In the BMP-2 signaling pathway, NfIc acts as a transducer between Runx2 and Osx, with NfIc directly regulating Osx expression and Runx2 upstream. 26 Osx is found in the enhancer region of primary osteoblasts and can form enhanceosomes with myocyte enhancer factor 2 and Dlx5 to activate the osteoblast-specific enhancer Runx2 in a synergistic manner, indicating that Osx also plays a role in controlling the expression of Runx2. This implies that Osx and Runx2 regulate each other in a manner akin to an indirect positive loop. 27

Numerous mature osteoblast genes, such as collagen type I a1, osteonectin, osteopontin, osteocalcin, and bone sialoprotein, which are necessary for productive osteoblasts during bone creation, will subsequently be activated by the Osx transcription factor. 23 The expression of Runx2 rises in direct proportion to that of ALP. The Runx2 activation of osteogenic differentiation results in increased ALP activity, osteoblast maturation, and the messenger ribonucleic acid expression of genes linked to osteogenesis, namely, ALP, collagen type I, and osteocalcin. ALP, collagen type I, osteocalcin, and osteopontin are among the genes involved in osteogenic differentiation whose promoter activity is regulated by the crucial regulator Runx2. During osteoblast development, Runx2 and ALP have a reciprocal regulatory interaction. 27

According to this study, β-TCP can promote the osteogenic differentiation of ADMSCs. This is in line with several other studies that have shown that β-TCP can increase the expression of ALP and osteocalcin in human MSCs 28 ; ALP, osteocalcin, and osteopontin in human adipose MSCs 29 ; Osx and osteocalcin in adipose MSCs 30 ; BMP-2 in RAW 264.7 cells; and ALP, osteocalcin, and Runx2 in bone marrow and adipose MSCs. 25 31 It has been shown that the β-TCP pore size used in this study affects the migration, osteogenic differentiation, and survival of ADMSCs. As demonstrated by the enhanced production of osteogenic markers, the study's β-TCP size of 300 to 600 µm provided better osteogenic differentiation results than the β-TCP size of 600 to 1,000 µm. The β-TCP particle size matches the one that is currently in use and available for purchase. 19 20 Nevertheless, there are several limitations in the present study: Alizarin red staining and calcium assay quantification were not investigated in this study to examine the osteodifferentiation of ADMSCs. In addition, this study only examined limited osteodifferentiation markers of ADMSCs after exposure to the β-TCP bioceramics' granule sizes.

## Conclusion

ADMSC osteodifferentiation was influenced by the β-TCP bioceramic granule size. The considerable difference in the expression of osteonectin and osteopontin supports the idea that 300 to 600 µm β-TCP exhibits better osteoinductivity than 600 to 1,000 µm β-TCP. The reason behind the notable variations observed solely in the late osteoblastogenesis biomarker is yet unknown. However, this study result may be beneficial as a foundation to develop β-TCP biomaterials, which may induce the osteodifferentiation of MSCs that can be used for bone defect tissue engineering. Therefore, more research is required to explain these findings using various experimental assessment techniques, in-depth analyses, and longer observation times.

## References

[1] Nugraha A P Yang H Chen J β-tricalcium phosphate as alveolar bone grafting in cleft lip/palate: a systematic review Dent J 2023 11 10 234 10.3390/dj11100234 PMC10606107 37886919

[2] Putri I L Fatchiyah Fatchiyah Pramono C Alveolar repair using cancellous bone and beta tricalcium phosphate seeded with adipose-derived stem cell Cleft Palate Craniofac J 2024 61 04 555 565 36237116 10.1177/10556656221132372

[3] Roy A-A Rtshiladze M A Stevens K Phillips J Orthognathic surgery for patients with cleft lip and palate Clin Plast Surg 2019 46 02 157 171 30851748 10.1016/j.cps.2018.11.002

[4] Putri I L Fabian P Wungu C DK A meta-analysis of alveolar bone grafting using bone substitutes in cleft lip and palate patients Tzu Chi Med J 2024 36 01 53 58 38406575 10.4103/tcmj.tcmj_125_23 PMC10887340

[5] Bohner M Santoni B LG Döbelin N β-tricalcium phosphate for bone substitution: synthesis and properties Acta Biomater 2020 113 23 41 32565369 10.1016/j.actbio.2020.06.022

[6] Özgenç Ö Özen A Osteogenic differentiation of canine adipose derived mesenchymal stem cells on B-TCP and B-TCP/collagen biomaterials Ankara Univ Vet Fak Derg 2024 71 02 125 134

[7] Putri T S Ratnasari A Sofiyaningsih N Nizar M S Yuliati A Shariff K A Mechanical improvement of chitosan–gelatin scaffolds reinforced by β-tricalcium phosphate bioceramic Ceram Int 2022 48 08 11428 11434

[8] Kasten P Beyen I Niemeyer P Luginbühl R Bohner M Richter W Porosity and pore size of β-tricalcium phosphate scaffold can influence protein production and osteogenic differentiation of human mesenchymal stem cells: an in vitro and in vivo study Acta Biomater 2008 4 06 1904 1915 18571999 10.1016/j.actbio.2008.05.017

[9] Putri T S Elsheikh M Flexural strength evaluation of chitosan-gelatin-B-tricalcium phosphate-based composite scaffold J Int Dental Med Res 2022 15 01 31 34

[10] Putri T S Sunarso Sunarso Hayashi K Tsuru K Ishikawa K Feasibility study on surface morphology regulation of β-tricalcium phosphate bone graft for enhancing cellular response Ceram Int 2022 48 09 13395 13399

[11] Rantam F A Nugraha A P Ferdiansyah F A potential differentiation of adipose and hair follicle-derived mesenchymal stem cells to generate neurons induced with EGF, FGF, PDGF and forskolin Res J Pharm Technol 2020 13 01 275 281

[12] Wicaksono S Nugraha A P Rahmahani J Adipose mesenchymal stem cell metabolites oral gel enhance pro-angiogenic factors expression, angiogenesis, and clinical outcome of oral ulcer rat model Eur J Dent 2024 18 01 117 123 36963426 10.1055/s-0043-1761192 PMC10959621

[13] Ferdiansyah Satuman Sari D S Maduratna E Latief F DE Nugraha A P Sudiana K Rantam F A Osteogenic differentiation and biocompatibility of bovine teeth scaffold with rat adipose-derived mesenchymal stem cells Eur J Dent 2019 13 02 206 212 31525778 10.1055/s-0039-1694305 PMC6777160

[14] Susanto H Ratu Mas Saraswati A Patera Nugraha A Wicaksono S Nur'aeny N Savitri Ernawati D Topical adipose mesenchymal stem cell metabolites regulate the expression of MMP-1, MMP-9, EGF, TGF-β in oral mucosa ulcer rat model Saudi Dent J 2024 36 06 932 939 38883902 10.1016/j.sdentj.2024.03.021 PMC11178951

[15] Prahasanti C Nugraha A P Saskianti T Suardita K Riawan W Ernawati D S Exfoliated human deciduous tooth stem cells incorporating carbonate apatite scaffold enhance BMP-2, BMP-7 and attenuate MMP-8 expression during initial alveolar bone remodeling in wistar rats (Rattus norvegicus) Clin Cosmet Investig Dent 2020 12 79 85 10.2147/CCIDE.S245678 PMC7102906 32273773

[16] Alhasyimi A A Suparwitri S Christnawati C Effect of carbonate apatite hydrogel-advanced platelet-rich fibrin injection on osteoblastogenesis during orthodontic relapse in rabbits Eur J Dent 2021 15 03 412 419 33368063 10.1055/s-0040-1721234 PMC8382455

[17] Nugraha A P Narmada I B Winoto E R Gingiva mesenchymal stem cells normoxic or hypoxic preconditioned application under orthodontic mechanical force on osterix, osteopontin, and ALP expression Eur J Dent 2024 18 02 501 509 37995729 10.1055/s-0043-1772699 PMC11132784

[18] Nugraha A P Narmada I B Ernawati D S Bone alkaline phosphatase and osteocalcin expression of rat's Gingival mesenchymal stem cells cultured in platelet-rich fibrin for bone remodeling ( *in vitro* study) Eur J Dent 2018 12 04 566 573 30369804 10.4103/ejd.ejd_261_18 PMC6178667

[19] Neamat A Gawish A Gamal-Eldeen A M β-Tricalcium phosphate promotes cell proliferation, osteogenesis and bone regeneration in intrabony defects in dogs Arch Oral Biol 2009 54 12 1083 1090 19828137 10.1016/j.archoralbio.2009.09.003

[20] Piccinini M Prosperi S Preve E Rebaudi A Bucciotti F In vitro biocompatibility assessment and in vivo behavior of a new osteoconductive βtCP bone substitute Implant Dent 2016 25 04 456 463 27455428 10.1097/ID.0000000000000442

[21] Nugraha A P Rantam F A Narmada I B Ernawati D S Ihsan I S Gingival-derived mesenchymal stem cell from rabbit (Oryctolagus cuniculus): isolation, culture, and characterization Eur J Dent 2021 15 02 332 339 33260232 10.1055/s-0040-1719213 PMC8184309

[22] Mohammed A HM Shariff K A Abu Bakar M H Mohamad H Fabrication of macro- and micropore forming DCPD/β-TCP porous scaffolds: effect of setting-time reaction and acidic calcium phosphate solution Iran J Mater Sci Eng. 2023 20 04 1 10

[23] Mohammed Mohammed A H Shariff K A Wahjuningrum D A Bakar M HA Mohamad H A comprehensive review of the effects of porosity and macro- and micropore formations in porous β-TCP scaffolds on cell responses J Aust Ceram Soc. 2023 59 04 865 879

[24] Mikai A Ono M Tosa I BMP-2/β-TCP local delivery for bone regeneration in MRONJ-like mouse model Int J Mol Sci 2020 21 19 7028 32987737 10.3390/ijms21197028 PMC7583034

[25] Chen Z Wu C Gu W Klein T Crawford R Xiao Y Osteogenic differentiation of bone marrow MSCs by β-tricalcium phosphate stimulating macrophages via BMP2 signalling pathway Biomaterials 2014 35 05 1507 1518 24268199 10.1016/j.biomaterials.2013.11.014

[26] Zhu S Chen W Masson A Li Y P Cell signaling and transcriptional regulation of osteoblast lineage commitment, differentiation, bone formation, and homeostasis Cell Discov 2024 10 01 71 38956429 10.1038/s41421-024-00689-6 PMC11219878

[27] Liu Q Li M Wang S Xiao Z Xiong Y Wang G Recent advances of Osterix transcription factor in osteoblast differentiation and bone formation Front Cell Dev Biol 2020 8(December):601224 33384998 10.3389/fcell.2020.601224 PMC7769847

[28] Choy C S Lee W F Lin P Y Surface modified β-tricalcium phosphate enhanced stem cell osteogenic differentiation in vitro and bone regeneration in vivo Sci Rep 2021 11 01 9234 33927241 10.1038/s41598-021-88402-5 PMC8084957

[29] Marino G Rosso F Cafiero G β-tricalcium phosphate 3D scaffold promote alone osteogenic differentiation of human adipose stem cells: in vitro study J Mater Sci Mater Med 2010 21 01 353 363 19655233 10.1007/s10856-009-3840-z

[30] Herrera D Lodoso-Torrecilla I Ginebra M P Rappe K Franch J Osteogenic differentiation of adipose-derived canine mesenchymal stem cells seeded in porous calcium-phosphate scaffolds Front Vet Sci 2023 10(June):1.149413E6 10.3389/fvets.2023.1149413 PMC10272761 37332740

[31] Park H Kim J S Oh E J Effects of three-dimensionally printed polycaprolactone/β-tricalcium phosphate scaffold on osteogenic differentiation of adipose tissue- and bone marrow-derived stem cells Arch Craniofac Surg 2018 19 03 181 189 30282427 10.7181/acfs.2018.01879 PMC6177683

